# The estrogen-progestogen-oxidative stress network in uterine fibroids: mechanistic insights and therapeutic opportunities

**DOI:** 10.1080/13510002.2026.2622747

**Published:** 2026-02-04

**Authors:** Siyu Wang, Wanhui You, Danni Ding, Fangyuan Liu, Fengjuan Han, Liping Tang

**Affiliations:** a Department of Obstetrics and Gynecology, Heilongjiang University of Chinese Medicine, Harbin, Heilongjiang Province, People’s Republic of China; b Department of Obstetrics and Gynecology, The First Affiliated Hospital of Heilongjiang University of Chinese Medicine, Harbin, Heilongjiang Province, People’s Republic of China; c Department of Obstetrics and Gynecology, The Affiliated Tumor Hospital of Harbin Medical University, Harbin, Heilongjiang Province, People’s Republic of China

**Keywords:** Uterine fibroids, estrogen, progesterone, oxidative stress, signaling network, molecular mechanisms, targeted therapy, combination therapy

## Abstract

Uterine fibroids are benign tumors with high incidence and recurrence rates that still pose significant treatment challenges. Traditionally, it has been believed that estrogen and progesterone primarily drive the development and progression of uterine fibroids. Recent studies have revealed that hormonal imbalance can affect reactive oxygen species production and trigger a significant oxidative stress (OS) state. The OS status in uterine fibroids can further amplify the pathological effects caused by hormonal imbalance. This suggests that estrogen, progesterone, and OS may interact to form an estrogen-progesterone-oxidative stress (E-P-OS) network, collectively promoting the progression of uterine fibroids. This network model provides a theoretical basis for the high recurrence rates following hormone monotherapy or surgery. Therefore, we reviewed the molecular mechanisms underlying hormone-OS interactions within the E-P-OS network and elucidated its pathological effects in promoting uterine fibroid progression. The integrated perspective lays the theoretical foundation for developing novel therapies that simultaneously block hormone signaling and counteract oxidative damage. Additionally, we summarized current clinical strategies for hormone therapy and antioxidant treatment, identified potential combination therapy approaches, and explored key challenges in their clinical translation. This aims to provide new directions and evidence for advancing the precision treatment of uterine fibroids.

## 1. Introduction

Uterine fibroids are the most common benign tumors of the female reproductive system. Epidemiological studies indicate that uterine fibroids are highly prevalent among women of reproductive age, with rates exceeding 70% in some regions [1]. In 2021, the age-standardized incidence rate and age-standardized prevalence rate for uterine fibroids were 250.93 and 2,841.07 per 100,000 people, respectively. These rates constitute a substantial global health burden [2]. The incidence of uterine fibroids is influenced by multiple factors, including ethnicity [3], body fat [4,5], and genetics [6]. Early uterine fibroids often present without noticeable symptoms, with most patients discovering them during consultations for heavy menstrual bleeding. As fibroids grow, they may cause pelvic compression symptoms such as frequent urination and constipation, and can even lead to severe anemia and infertility [7]. These clinical symptoms affect patients’ quality of life to varying degrees. Total hysterectomy is the preferred treatment option for patients with massive fibroids or severe symptoms. While this procedure offers a definitive cure, it permanently eliminates fertility, causing significant physical and psychological trauma to the patient [8]. Patients who wish to preserve their fertility may undergo a myomectomy, a procedure that allows the uterus to be preserved. However, the recurrence rate within five years of surgery is as high as 50% [9,10]. Non-invasive techniques, including high-intensity focused ultrasound, also face the challenge of recurrence [11]. More effective treatment approaches need to be explored to overcome current therapeutic limitations.

Uterine fibroids are widely recognized as hormone-dependent tumors, primarily influenced by estrogen and progesterone. Molecular pathology studies confirm that fibroid tissue exhibits significantly elevated expression of estrogen receptors (ER) and progesterone receptors (PR) [12]. Its high prevalence during the reproductive years and rapid increase during pregnancy fully confirm the central regulatory role of estrogen and progesterone [13]. Drug therapy, as a non-invasive treatment approach, is suitable for patients undergoing conservative management. Hormonal therapies, represented by gonadotropin-releasing hormone (GnRH) analogues and selective progesterone receptor modulators (SPRMs), constitute the mainstream clinical medications. While this method can significantly suppress fibroids and alleviate symptoms, rapid recurrence occurs after discontinuation of treatment [14]. Therefore, hormone therapy is effective to a certain extent but fails to address the root cause, strongly suggesting that the development of uterine fibroids involves other key drivers beyond traditional hormonal regulation [15].

Oxidative stress (OS) has been demonstrated to be widely prevalent in malignant tumors, playing a pivotal role in cancer progression by affecting genomic stability and the cellular microenvironment [16]. Similarly, studies of uterine fibroids provide direct evidence of a significant state of OS at the lesion site. Compared to adjacent normal myometrium, fibroid tissue exhibits markedly elevated levels of oxidative damage products alongside reduced activity of key antioxidant enzymes [17,18]. This localized OS environment has been demonstrated to contribute to the abnormal proliferation of uterine leiomyoma cells by inducing dysregulation in the expression of genes associated with proliferation and apoptosis [19,20]. These findings indicate that local OS represents an active pathological feature in established uterine fibroids, participating in abnormal cellular proliferation by regulating gene expression. Therefore, the pathological progression of uterine fibroids not only exhibits classic hormone dependence but also incorporates pathological features maintained and exacerbated by locally sustained OS. Consequently, the promoting effects of estrogen, progesterone, and OS on uterine fibroids have been extensively studied. However, the molecular mechanisms underlying their interactions remain a core issue that has not been fully elucidated within the field.

Based on this, we comprehensively elucidate the potential interplay between estrogen, progesterone, and OS in uterine fibroids. This interaction may form an estrogen-progesterone-oxidative stress (E-P-OS) network, maintaining the sustained growth of uterine fibroids. The establishment of the E-P-OS network offers a new perspective for understanding the high recurrence rate of uterine fibroids following single-hormone therapy or surgical intervention. Therefore, we will organize the direct and indirect evidence supporting the interactions between estrogen, progesterone, and OS around the integrated E-P-OS network mechanism. Critically evaluate the strengths and limitations of this evidence. Further elucidate the pathological outcomes resulting from this network in the development and progression of uterine fibroids. Building upon this foundation, we further evaluate novel therapeutic strategies targeting this network, particularly the feasibility of enhancing treatment efficacy through combined blockade of hormonal signaling and oxidative damage. Finally, we outline future research directions, offering new insights for both basic experimental and clinical studies aimed at treating uterine fibroids.

## 2. The case for the E-P-OS network framework

### 2.1. Hormonal imbalance of estrogen dominance and progesterone synergy

**Figure 1.**
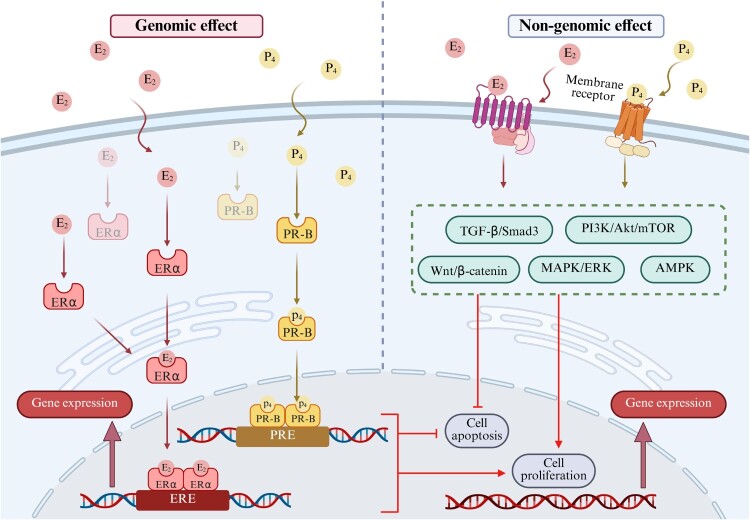
The action pathways of estradiol and progesterone in uterine fibroid cells. In uterine fibroid cells, overexpression of estrogen receptor alpha (ERα) and progesterone receptor-B (PR-B) constitutes a key pathological feature. Estradiol (E₂) primarily activates both genomic and non-genomic signaling pathways by binding to its overexpressed receptor ERα [22]. In the genomic pathway, the E₂-ERα complex is transported into the cell nucleus to regulate the transcription of target genes, including cyclins and growth factors. In the non-genomic pathway, E₂ rapidly activates downstream kinase signaling cascades via membrane-associated receptors. Progesterone (P₄) primarily exerts its biological effects through its overexpressed receptor PR-B, acting via both genomic and non-genomic mechanisms [23]. The synergistic interaction between E₂ and P₄ through their receptor signaling networks enables efficient signal transduction and amplification. This establishes the molecular foundation for the E-P-OS network and constitutes the initial driving core of this interaction network. Created in BioRender.

An imbalance between estrogen and progesterone constitutes a key initiating factor for uterine fibroids [21]. This imbalance primarily manifests as relative or absolute estrogen dominance coupled with enhanced progesterone action, which together establish the environmental foundation for abnormal proliferation of fibroid cells. This establishes the foundation for the E-P-OS network integration framework (Figure 1).

The incidence of uterine fibroids is positively correlated with the duration of endogenous estrogen exposure [24]. Pregnancy or exogenous estrogen supplementation can stimulate their growth, while fibroids typically shrink after menopause [25]. Sex hormones play a central role in the development of uterine fibroids, with estrogen concentrations being significantly higher in fibroid tissue than in normal myometrial tissue [26]. This localized hyperestrogenic state can be attributed to higher aromatase expression levels in fibroid tissue compared to normal myometrium, promoting the conversion of androstenedione to estrone (E_1_). E_1_ is then reduced to the more biologically active estradiol (E_2_) by the action of 17β-hydroxysteroid dehydrogenase (17β-HSD) type 1 [27]. In the biosynthesis of E_2_, cytochrome P450 (CYP) 17A1 and CYP19A1 are key enzymes. The metabolism of E_2_ primarily involves CYP1A1 and CYP1B1. Genetic variations in these key enzymes may collectively contribute to elevated estrogen levels in uterine fibroid tissue by enhancing E_2_ synthesis and altering the balance of E_2_ metabolic pathways [28,29]. Furthermore, studies have confirmed that uterine fibroid cells commonly overexpress ER, with ERα expression consistently higher than ERβ [30]. The expression level of ERα mRNA in uterine fibroids is 1.8- to 2.6-fold higher than that in adjacent normal myometrium [26]. Overexpression of ERα significantly amplified the sensitivity of uterine leiomyoma cells to circulating and local E_2_ signaling. High levels of E₂ bind to the overexpressed ERα, promoting tumor development through both genomic and non-genomic signaling pathways [22]. This directly activates the downstream Wnt/β-catenin, phosphatidylinositol 3-kinase/protein kinase B/mechanistic target of rapamycin (PI3 K/Akt/mTOR), mitogen-activated protein kinase/extracellular signal-regulated kinase (MAPK/ERK), and AMP-activated protein kinase (AMPK) signaling pathways, promoting the expression of Cyclin D1 and cellular myelocytomatosis (c-Myc) in fibroid tissue [31–33]. Crucially, E_2_ drives the expression of multiple growth factors, including insulin-like growth factor 1 (IGF-1) and transforming growth factor-β (TGF-β). This significantly stimulates the abnormal synthesis and deposition of major extracellular matrix (ECM) components [32,34,35].

The role of progesterone in fibroid growth is not merely that of an estrogen antagonist, but rather it actively exhibits a synergistic effect. Particularly during the luteal phase or under the influence of exogenous progestogens, it promotes mitotic effects and strongly stimulates the proliferation of fibroid cells [36]. Research confirms that high-dose progesterone upregulates Ki67 mRNA expression [37] and synergistically enhances the expression of proliferating cell nuclear antigen (PCNA), epidermal growth factor (EGF), and epidermal growth factor receptor (EGFR) with E_2_, thereby enhancing the proliferative potential of fibroid cells [38]. It also upregulates the expression of the anti-apoptotic protein B-cell lymphoma 2 (Bcl-2), thereby inhibiting apoptosis [38]. High concentrations of E_2_ in uterine fibroid tissue induce progesterone receptor expression, predominantly of the PR-B subtype, by binding to ERα. This overexpression ultimately leads to heightened progesterone sensitivity in fibroid cells [39,40]. Progesterone primarily activates genomic and non-genomic signaling pathways by binding to the PR-B [23]. These pathways extensively interact with E_2_-activated Wnt/β-catenin, PI3 K/Akt/mTOR, and MAPK/ERK signaling pathways. By enhancing the phosphorylation levels of key molecules in E_2_ signaling pathways, they form positive feedback loops that further amplify the effects of E_2_ [33,41,42]. Additionally, progesterone independently and potently drives abnormal deposition and remodeling of the ECM, which is key to the enlargement and hardening of fibroids. Progesterone rapidly activates the TGF-β signaling pathway and its downstream effector molecule Smad3 [43]. Promotes the synthesis and deposition of type I collagen, fibronectin (FN), versican, and dermatopontin in the ECM, while simultaneously suppressing the expression of decorin, which possesses anti-fibrotic effects [44,45]. This potent stimulation of ECM synthesis acts synergistically with the proliferative effects of E₂, collectively driving the uncontrolled growth of uterine fibroids.

Therefore, the core mechanism of ‘hormonal imbalance’ in uterine fibroids lies in the dominant driving role of E_2_ and the synergistic amplifying effect of progesterone. Its essence lies in a complex pathological network that is driven by the overexpression of ERα and PR-B. This receptor-driven hormone network directly promotes the proliferation and fibrosis of uterine fibroids, and establishes the conditions that influence local OS. This initiates the entire E-P-OS network.

### 2.2. Hormonal modulation of oxidative stress

**Figure 2.**
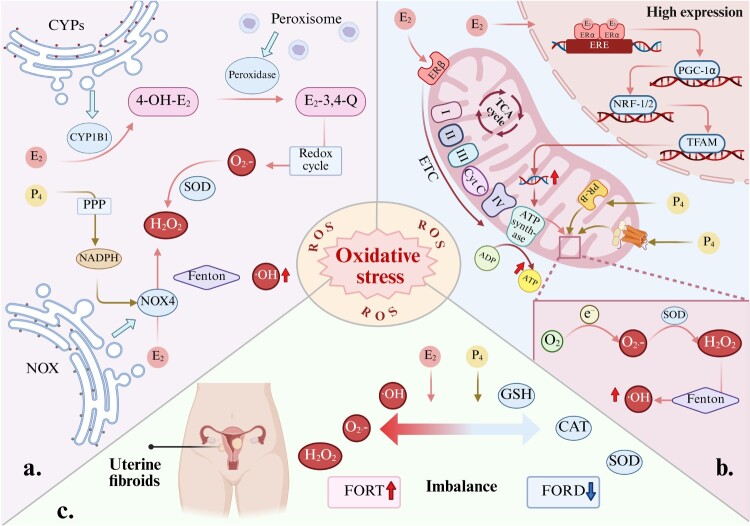
Three potential pathways by which E2 and progesterone regulate OS in uterine fibroids. (a) Enzymatic metabolic imbalance: E₂ and progesterone induce ROS accumulation by regulating the expression of enzymes such as CYP1B1 and NOX4 [46,47]. (b) Mitochondrial dysfunction: E₂ synergistically with progesterone induces hyperactive mitochondrial biogenesis and impaired electron transport chain function, thereby promoting reactive oxygen species (ROS) production [48–51]. (c) Antioxidant System Suppression: E₂ and progesterone interfere with the expression and activity of key antioxidant enzymes such as superoxide dismutase (SOD) and catalase (CAT), while depleting glutathione (GSH) [18,52]. Created in BioRender.

The hormonal imbalance centered on E₂ not only promotes reactive oxygen species (ROS) production but also weakens the cell's antioxidant defense capabilities. Progesterone may further amplify this effect synergistically, collectively leading to an imbalance in intracellular redox homeostasis. Evidence from multiple fields, including metabolism, cell biology, and clinical endocrinology, collectively suggests that within the uterine fibroid microenvironment, E_2_ and progesterone may induce excessive ROS accumulation through three pathways: enzyme metabolic imbalance, mitochondrial dysfunction, and disruption of the antioxidant system. This perpetually maintains fibroids in a state of OS (Figure 2).

#### 2.2.1. Enzyme metabolic imbalance

The metabolism of E₂ within uterine fibroid cells represents a potential mechanism for its induction of OS. Research indicates that uterine fibroid tissue exhibits abnormally high expression of CYP1B1, an enzyme responsible for hydroxylating E₂ into the highly active metabolite 4-hydroxyestradiol (4-OH-E₂). This metabolite is then primarily oxidized by peroxidase into estradiol-3,4-quinone [46,53,54]. Although this metabolic pathway has been more extensively studied in tissues such as the liver, the unique high CYP1B1 environment in fibroid tissue suggests that local metabolism of E₂ may constitute an intrinsic source of ROS [46]. This quinone compound continuously generates superoxide anion (O₂•^−^) through redox cycling, which is catalyzed by superoxide dismutase (SOD) to produce hydrogen peroxide (H₂O₂). Subsequently, via the Fenton reaction, it ultimately generates highly toxic hydroxyl radicals (•OH) [55,56]. Therefore, the metabolic activation of E₂ may represent a self-amplifying mechanism that initiates and sustains the local OS state in uterine fibroids [46].

E₂ may also directly generate ROS by activating the NADPH oxidase (NOX) family, particularly NOX4 [57,58]. Importantly, the high expression of NOX4 in uterine fibroid tissue has been experimentally confirmed [47]. Based on this, E₂ may generate H₂O₂ in uterine fibroids by upregulating the expression and activity of NOX4 in the endoplasmic reticulum. H₂O₂ not only causes oxidative damage but also acts as a second messenger to activate multiple downstream pro-growth signaling pathways [59]. Additionally, progesterone promotes nicotinamide adenine dinucleotide phosphate (NADPH) production in uterine epithelial cells via the pentose phosphate pathway [60]. This provides NOX4 with the essential electron donor for its catalytic reaction. Based on the evidence that NOX4 is highly expressed in uterine fibroid tissues [47], it can be inferred that progesterone can provide auxiliary support for E₂-induced NOX4 activity in fibroid cells through a similar mechanism, thereby potentially synergistically amplifying the pro-oxidative effect of E₂ functionally. However, this speculation needs to be directly verified in uterine fibroid cell models.

#### 2.2.2. Mitochondrial dysfunction

Mitochondria serve as the cellular powerhouses, and their dysfunction is linked to a self-reinforcing vicious cycle involving excessive ROS production [61,62]. This cycle is particularly pronounced in proliferative cells with high energy demands. Uterine fibroid cells exhibit active mitochondrial biogenesis, which is considered fundamental to supporting their rapid proliferation [63]. E₂ may be the key driver of this phenomenon. Research indicates that E₂ binds to ER within the cell nucleus, activating the expression of peroxisome proliferator-activated receptor gamma coactivator 1-alpha (PGC-1α). Activated PGC-1α subsequently activates nuclear respiratory factor 1 (NRF-1) and NRF-2, thereby stimulating the expression of related factors such as mitochondrial transcription factor A (TFAM). This ultimately leads to increased mitochondrial DNA replication, elevated cellular mitochndrial numbers, and significantly enhanced mitochondrial biogenesis capacity [64–66]. Additionally, evidence indicates that E₂ can also directly enhance the activity of the electron transport chain (ETC) by binding to ERβ within mitochondria, thereby improving cellular respiration and ATP production [48,49]. Considering these mechanisms within the pathological context of high E₂ and ER expression in uterine fibroids, it can be concluded that E₂ may contribute to mitochondrial hyperfunction through the aforementioned pathways. Studies in cellular models have revealed that progesterone can bind to receptors located on mitochondria, leading to elevated membrane potential and a surge in oxygen consumption rate [50]. Although this study was not conducted in fibroid cells, it suggests that progesterone may participate in ROS generation by affecting mitochondrial function. The specific mechanism of action in uterine fibroids still requires direct experimental verification, but this hormone-driven, hyperactive state of the electron transport chain is considered the primary source of increased electron leakage, leading to massive ROS production [51,56]. Ultimately, these excessively produced ROS collectively contribute to the proliferation and progression of fibroids.

#### 2.2.3. Disruption of the antioxidant system

Under physiological conditions, the body's antioxidant system can promptly eliminate excess ROS. However, in uterine fibroid cells, this balance is disrupted [67]. Direct experimental evidence indicates that compared to normal uterine myometrial cells, both SOD and CAT mRNA levels and enzyme activity in uterine fibroid cells are significantly reduced. This suggests a severe functional defect in the antioxidant enzyme system of fibroid cells [18]. In addition to the impairment of the antioxidant enzyme system, the non-enzymatic antioxidant defense system may also be affected. A study suggests that excessive E_2_ can interfere with the activity of key enzymes involved in glutathione (GSH) metabolism, thereby affecting the synthesis and regeneration of this vital antioxidant [52]. A study in a rat uterine fibroid model demonstrated that reducing serum E_2_ levels was accompanied by increased GSH content, thereby alleviating the extent of oxidative damage [68]. Based on this, it is reasonable to conclude that the high E_2_ environment associated with uterine fibroids can further compromise the antioxidant defense capacity of cells [69]. A clinical observational study provides indirect support for this view. Research has found that in women receiving combined high-dose E₂ and progesterone therapy, the ratio of OS markers (FORT) to antioxidant capacity markers (FORD) significantly increased. This indicates that combined progesterone and E₂ therapy is associated with elevated OS indicators, suggesting it may contribute to worsening OS status [70]. Based on the available evidence, the unique hormonal environment within uterine fibroids may collectively undermine the cellular antioxidant defense network by directly diminishing antioxidant enzyme activity and disrupting the non-enzymatic antioxidant system. The persistent overproduction of ROS within cells, coupled with severely impaired clearance mechanisms, disrupts intracellular redox homeostasis. This establishes and sustains a chronic state of OS conducive to the growth and progression of uterine fibroids.

### 2.3. Oxidative stress as a potential modulator of hormonal signaling

OS may modulate the biological effects of E₂ and progesterone through multiple pathways, with its primary mechanisms involving two aspects: synergistic effects with hormone signaling in key pathways, and potential influence on hormone receptor expression and function. These findings provide a theoretical basis for elucidating the role of OS as a potential regulator of hormone signaling.

#### 2.3.1. Synergistic effects of hormone signaling pathways

**Figure 3.**
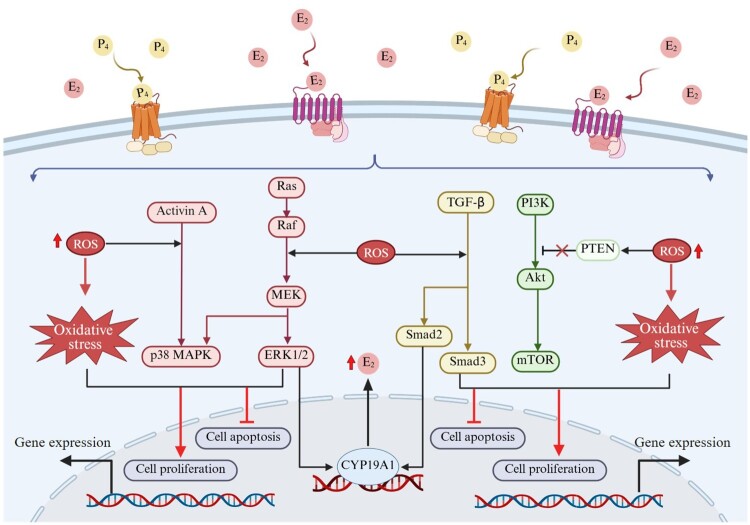
Potential molecular mechanisms by which OS enhances the pathological effects of hormone signaling pathways on uterine fibroids. The accumulation of ROS not only directly leads to OS status, but also potentially regulates the TGF-β/Smad3, MAPK, and PI3 K/Akt/mTOR signaling pathways, which are activated by E₂ and progesterone through non-genomic pathways. ROS can influence the balance between cell proliferation and apoptosis through interactions with these signaling pathways, thereby participating in the pathological progression of uterine fibroids. Created in BioRender.

OS can activate TGF-β/Smad3, PI3 K/Akt/mTOR, and MAPK signaling pathways, which are also key downstream pathways for E₂ and progesterone signaling. This cross-talk mechanism among signaling pathways provides a theoretical basis for understanding OS as a potential modulator of hormone signaling (Figure 3).

The TGF-β/Smad3 pathway is primarily regulated by progesterone during the fibrosis process of uterine fibroids [43,71]. In uterine fibroids, the regulation of this pathway by progesterone may also be modulated by OS. A study on uterine fibroid cells revealed that a key component of this pathway involves the overexpression of NOX4, leading to excessive ROS production [72]. Notably, studies in cancer cells indicate that ROS can form a positive feedback regulation with TGF-β. This mechanism may also be present in uterine fibroids, but further experimental validation is required [73]. Based on existing evidence, the potential interaction between ROS and TGF-β may sustain activation of the TGF-β/Smad3 pathway, thereby enhancing progesterone-mediated pro-fibrotic and proliferative effects [43,72]. Furthermore, it is worth exploring whether the activated TGF-β/Smad signaling pathway may participate in the pathological process of uterine fibroids by regulating E₂ metabolism. Research indicates that in human granulosa cells, TGF-β1 can upregulate CYP19A1 expression via the Smad2 and ERK1/2 signaling pathways, thereby promoting E₂ production [74]. It should be noted that whether this mechanism also exists in uterine fibroids remains to be confirmed, but it provides a promising avenue for exploring the localized high E₂ state in fibroids.

Activation of the MAPK pathway participates in ECM synthesis and fibrosis in fibroid tissue [71]. The accumulation of ROS has been demonstrated to trigger the phosphorylation and activation of the key kinase p38 MAPK, suggesting that ROS is a potential activator of the MAPK signaling pathway [75]. In the context of uterine fibroids, the Activin A-p38 MAPK signaling axis has been demonstrated to be a key molecular mechanism mediating excessive ECM production in fibroid tissue and driving its growth [76]. These findings suggest that OS may participate in the fibrotic process of myomas by intervening in nodes such as p38 MAPK. E₂ and progesterone are both key regulators of the MAPK pathway. Studies indicate that in uterine fibroid cells, high concentrations of E₂ can rapidly activate the Ras-Raf-MEK-ERK signaling cascade via a non-genomic pathway, thereby promoting cell proliferation [77,78]. Simultaneously, the pro-fibrotic effect of progesterone is also closely associated with the activation of the p38 MAPK pathway [79]. OS may exhibit synergistic effects with these hormonal signals, jointly regulating the activity of the MAPK pathway.

The PI3 K/Akt/mTOR signaling pathway plays a crucial role in regulating cell growth and proliferation. [80]. In uterine fibroids, dysfunction of MnSOD has been demonstrated to cause substantial accumulation of ROS and is associated with abnormal activation of the Akt signaling pathway [81]. This finding suggests that OS may participate in the regulation of this pathway by affecting the antioxidant enzyme system. Additionally, studies indicate that H₂O₂-mediated ROS can inactivate PTEN through oxidation, which is considered one of the potential mechanisms affecting the activity of the PI3 K/Akt/mTOR pathway [82]. Notably, E₂ and progesterone, as key regulators of uterine fibroids, have been demonstrated to activate the PI3 K/Akt pathway and promote the proliferation of fibroid cells [83]. Concurrently, IGF-1, as a crucial synergistic factor for E_2_ and progesterone, has been demonstrated in uterine fibroid studies to activate this pathway by upregulating the phosphorylation levels of PI3 K, Akt, and mTOR. This subsequently induces the expression of key cell cycle regulatory proteins, thereby participating in the abnormal proliferation process of fibroid cells [84]. These findings suggest that OS may act as a modulator, synergistically modulating the activation state of the PI3 K/Akt/mTOR pathway in conjunction with hormonal signaling.

Based on the above evidence, within the uterine fibroid microenvironment dominated by E_2_ and progesterone, OS may exert synergistic effects with hormonal signaling by modulating the activation status of key signaling pathways such as TGF-β/Smad3, PI3 K/Akt/mTOR, and MAPK, collectively forming a complex pro-growth signaling network. It is important to emphasize that current evidence is limited to the potential association between OS and hormone signaling within these pathways. The specific molecular mechanisms by which OS modulates hormone signaling, as well as its effects on other key signaling pathways such as Wnt/β-catenin and AMPK, remain to be further explored.

#### 2.3.2. Modulate the function of hormone receptors

A study found that antioxidant enzyme activity in uterine fibroid tissue was negatively correlated with ER and PR expression levels [85]. Animal model studies have also demonstrated that under stress conditions, ER and PR expression increase and correlate positively with OS levels [86]. Additionally, a phosphoproteomics study on uterine fibroids revealed that OS significantly alters the phosphorylation status of a series of key signaling proteins within fibroid tissue. This disruption in post-translational modifications may indirectly affect the activity of ER or PR [87]. However, this study did not directly validate whether ER or PR themselves serve as terminal targets for phosphorylation alterations induced by OS.

Although the above studies have revealed the association between OS and the function of hormone receptors, the molecular mechanisms by which OS regulates the transcription, translation, or specific post-translational modifications of ER and PR remain unclear. This knowledge gap can be significantly enlightened by relevant studies on other hormone-dependent diseases. In breast cancer, studies indicate that OS can upregulate the expression or enhance the activity of ER and PR by activating signaling pathways such as PI3 K/Akt/mTOR and MAPK/ERK [88,89]. This process involves a phosphorylation cascade of key kinases [89], which mechanistically corresponds to the phosphoproteomic alterations observed in uterine fibroids [87]. This suggests that OS may broadly affect receptor function by disrupting the kinase-phosphatase equilibrium. Additional research suggests that disruption of redox homeostasis may impair the function of key enzymes involved in estrogen metabolism, leading to the accumulation of genotoxic metabolites. This process may subsequently upregulate ER expression and enhance its stability by activating stress-related signaling pathways [90]. Furthermore, a review on gynecological diseases suggests that OS can directly interfere with hormone receptor function, while the application of antioxidants helps restore normal receptor signaling. This indirectly corroborates the pivotal role of OS in receptor regulation [91].

In summary, evidence supporting hormonal imbalance as a fundamental driver of OS status is relatively robust. However, the amplifying effect of OS on intracellular hormonal signaling in uterine fibroid cells requires further direct evidence to substantiate. However, integrating current direct and indirect evidence suggests that the interaction between hormones and OS may constitute a self-sustaining E-P-OS network, which collectively maintains the persistent growth of fibroids.

## 3. Downstream pathological effects driven by the E-P-OS network in uterine fibroids

**Figure 4.**
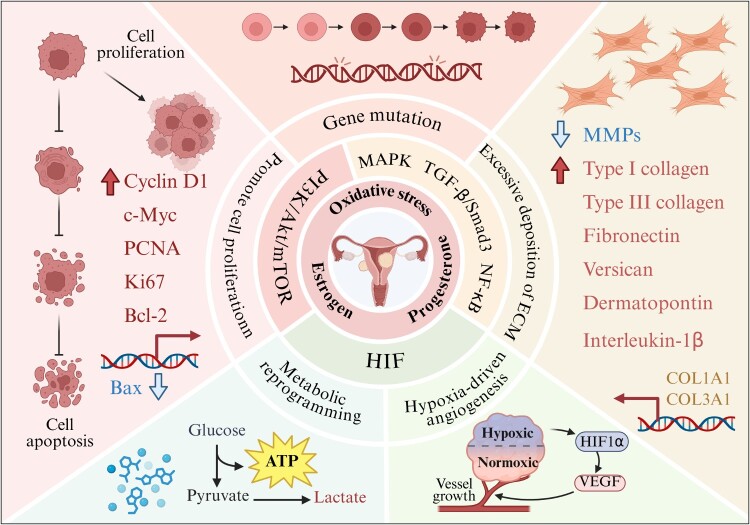
Pathological effects of the E-P-OS network on uterine fibroids. Created in BioRender.

Persistent hormonal imbalance and the OS state within the E-P-OS network promote the development of uterine fibroids not only by directly promoting cell proliferation, inhibiting apoptosis, and enhancing the fibrotic process. More significantly, this persistent state may also triggers deeper downstream effects. These effects include inducing tumor microenvironment remodeling, promoting tissue hypoxia, driving metabolic reprograming in fibroid cells, and even increasing genomic instability to facilitate gene mutations (Figure 4).

### 3.1. Promote cell proliferation and inhibit apoptosis

The E-P-OS network activates pro-survival and proliferation pathways such as PI3 K/Akt/mTOR, leading to the overexpression of Cyclin D1, c-Myc, PCNA, and Ki67 in uterine fibroid cells. This enables cells to successfully pass through the G1/S checkpoint and enter the S phase of the cell cycle, thereby initiating DNA synthesis and cell division [31,32,37]. Simultaneously, activation of mTOR promotes the provision of essential material foundations for cell proliferation [92]. The E-P-OS network may inhibit the pro-apoptotic pathway while simultaneously activating the pro-proliferative pathway. In normal cells, there is a delicate balance between the pro-apoptotic protein Bax and the anti-apoptotic protein Bcl-2 [93]. Together with members of the caspase family, this equilibrium regulates apoptosis homeostasis [94]. The E-P-OS network may contribute to this anti-apoptotic state. By suppressing Bax expression and promoting the expression of anti-apoptotic proteins such as Bcl-2, it strongly inhibits caspase cascade activity, thereby significantly enhancing the resistance of fibroid cells to apoptotic signals. Targeted inhibition of key nodes in the E-P-OS network may effectively reverses the anti-apoptotic phenotype and restores cellular sensitivity to apoptotic signals [95]. These molecular mechanisms act together to cause fibroid cells to proliferate abnormally rapidly and to become less sensitive to apoptotic signals. This ultimately drives the continuous enlargement of fibroids. Clinically, detecting the expression levels of proliferation markers such as PCNA and Ki-67 in fibroid tissue provides a direct reflection of cellular proliferation activity. In the pathological tissue of uterine fibroid patients, the positive expression rates of PCNA and Ki-67 are significantly elevated [96]. Further evidence demonstrates the pivotal role of the E-P-OS network in accelerating cell proliferation and inhibiting apoptosis within the pathogenesis of uterine fibroids.

### 3.2. Excessive deposition of ECM and fibrosis

E-P-OS network exerts a potential coordinating role in the processes of excessive ECM deposition and fibrosis by integrating hormone and OS signaling. This network activates the TGF-β/Smad3 and MAPK pathways to promote fibrosis in uterine fibroids [71]. Under the regulation of this pathological network, the levels of FN, versican, dermatopontin, and type I and III collagen in the ECM are significantly elevated in uterine fibroid tissue [44,97]. Concurrently, increased expression of tissue inhibitor of metalloproteinase-1 (TIMP-1) within uterine fibroid tissue inhibits matrix metalloproteinases (MMPs) activity, resulting in reduced degradation of the ECM and exacerbating its excessive deposition [98]. The E-P-OS network may also promote myoma fibrosis by inducing inflammatory responses. Studies indicate that in uterine fibroids, interleukin-1β concentrations correlate positively with nuclear factor kappa-light-chain-enhancer of activated B cells (NF-κB) levels, suggesting that inflammatory factors drive inflammatory responses and the fibrotic process by activating the NF-κB signaling pathway [99]. Abnormal deposition of large amounts of ECM components significantly increases the hardness of uterine fibroids, which is the core reason for their firm texture. Microscopic examination of fibroid tissue sections reveals numerous disorganized collagen fibers alongside increased numbers of fibroblasts and myofibroblasts. These pathological changes further confirm the pivotal role of excessive ECM deposition and fibrosis in the pathogenesis of uterine fibroids [100].

### 3.3. Hypoxia-driven angiogenesis

The E-P-OS network mediates rapid proliferation and fibrosis in uterine fibroids, with this abnormally accelerated growth significantly increasing local tissue demand for oxygen and nutrients [43]. When angiogenesis lags behind cellular proliferation, relative hypoxia develops, forming a hypoxic microenvironment. Hypoxia further enhances the proliferative activity and fibrotic phenotype of myoma cells by activating hypoxia-inducible factor (HIF) [19,101]. However, this does not imply that it possesses the invasive or metastatic capabilities of malignant tumors; the pathological changes remain confined to the realm of benign proliferation. This series of pathological alterations further exacerbates local hypoxia and, through the classic hypoxia signaling pathway, stabilizes HIF-1α, thereby driving disease progression [102]. Studies indicate that under hypoxic conditions, the degradation of HIF-1α is inhibited, causing it to accumulate in the cytoplasm. HIF-1α then translocates to the nucleus, where it binds to HIF-1β to form heterodimers. These complexes function as transcription factors that further localize to the nucleus and activate the expression of pro-angiogenic genes [103]. ISHIKAWA et al. demonstrated by Western blot analysis that nuclear extracts from uterine fibroid tissues exhibited significantly higher HIF-1α protein expression levels compared to those from adjacent myometrial tissues. Immunopositivity for HIF-1α was observed in cellular components of both uterine fibroids and the surrounding myometrium. Hypoxia increases HIF-1α protein levels while inducing vascular endothelial growth factor-A (VEGF-A) mRNA expression [101]. As a key pro-angiogenic factor, abnormal VEGF-A secretion may promote angiogenesis in uterine fibroid tissues, providing nutritional support that accelerates their proliferation. IYASHITA-ISHIWATA et al. reached similar conclusions through further research, demonstrating that hypoxia induces VEGF-A secretion in uterine fibroid cells [19]. Additionally, adrenomedullin, endothelin-1, and PCNA exhibit high expression levels in uterine fibroid tissue. This mechanism has not been observed in normal uterine myometrial cells, demonstrating that fibroid cells can continue to proliferate and promote angiogenesis under hypoxic conditions [19]. Notably, HIF-1α serves as the pivotal hub linking hypoxia to the E-P-OS network. By inducing TGF-β3 expression and activating the Smad3 pathway, it subsequently upregulates NOX4 and promotes ROS production, ultimately achieving positive reinforcement of E-P-OS network activity [72]. The hypoxic microenvironment promotes angiogenesis within fibroid tissue, yet the resulting blood vessels exhibit abnormally thin walls. Devoid of the multi-layered smooth muscle and elastic fibers characteristic of normal vessels, these abnormal vessels not only fail to effectively alleviate hypoxia but exacerbate tissue edema through increased vascular leakage. The leakage and rupture of these abnormal vessels directly disrupt the blood supply balance to the endometrium, leading to prolonged menstrual periods, increased menstrual flow, and even abnormal bleeding outside the menstrual cycle. This is precisely the core reason why abnormal uterine bleeding frequently accompanies uterine fibroids in clinical practice [104].

### 3.4. Metabolic reprogramming

Myoma cells undergo metabolic reprogramming to adapt to the hypoxic microenvironment maintained by the E-P-OS network. From an energy metabolism perspective, these cells preferentially utilize glycolysis for energy production. Although this pathway generates relatively less ATP, it offers faster reaction rates, enabling rapid energy supply for energy-intensive processes like cell division within short timeframes. This metabolic preference bears some resemblance to the Warburg effect observed in malignant tumors [105]. However, as benign tumors, uterine fibroids undergo metabolic reprogramming to support rapid proliferation rather than invasion. Under hypoxic conditions, pyruvate, a key glycolytic metabolite, is unable to efficiently enter the tricarboxylic acid cycle for complete oxidative metabolism. Instead, it is preferentially converted to lactate via anaerobic respiration pathways [106]. The accumulated lactic acid within fibroid tissue creates an acidic environment that suppresses immune cell function, weakening their ability to recognize and eliminate fibroid cells. This promotes immune evasion, thereby fostering a favorable environment for fibroid growth [107]. It is worth emphasizing that the hypoxia microenvironment induced by the E-P-OS network itself can directly activate the transcription of glycolysis-related genes such as glucose transporter 1 and lactate dehydrogenase A via HIF-1α, thereby further enhancing glycolysis and ensuring energy supply for fibroid cells under hypoxic conditions [108]. In summary, this glycolysis-centered metabolic reprogramming not only provides essential energy for the abnormal proliferation of leiomyoma cells but also influences the physiological state of surrounding tissues by altering the metabolic microenvironment (such as acidification and lactate signaling), constituting a key mechanism for the sustained growth of uterine fibroids.

### 3.5. Gene mutation

E-P-OS network promotes rapid proliferation of fibroid cells, with genetic mutations being the primary cause. As a core driver, E_2_ significantly shortens the DNA replication cycle by accelerating cell cycle progression, a process that directly increases the probability of DNA replication errors [109]. The metabolite 4-OH-E₂ exhibits direct genotoxicity by inducing DNA strand breaks or base oxidation. Furthermore, 4-OH-E₂ undergoes further metabolism to E₂-3,4-Q, a process that concurrently generates ROS. The accumulation of ROS subsequently triggers DNA damage [54]. Under the condition of continuous activation of the E-P-OS network, ROS-induced DNA damage persists. This ROS-triggered base modification not only alters the spatial conformation of DNA but also significantly elevates the base mismatch rate during DNA replication [110]. This widespread genomic instability provides the basis for the high-frequency, specific driver gene mutations observed in uterine fibroids. Notably, studies have begun to directly establish molecular links between oxidative damage and the characteristic mutations found in uterine fibroids. MED12 and HMGA2 are common mutation types in uterine fibroids, with MED12 mutations being the most prevalent [111]. Research confirms that in uterine fibroid tissue, the accumulation level of the oxidative damage marker 8-OHdG shows a significant positive correlation with MED12 mutation status, suggesting that an OS environment may represent a key selective pressure driving MED12 hotspot mutations [112]. Statistics indicate that 70% of uterine fibroid patients have MED12 mutations. These MED12 mutations further promote the expression of ECM-related genes, thereby accelerating fibroid fibrosis [113]. Meanwhile, experiments have shown that OS can induce genomic instability in uterine smooth muscle cells and specifically increase the probability of rearrangement in the HMGA2 gene [114,115]. It is undeniable that the genetic mutations observed in uterine fibroids typically confer a proliferative advantage to cells. However, these mutations do not endow cells with invasive or metastatic capabilities, representing one of the key distinctions between fibroids and malignant tumors. Overall, the persistent OS environment maintained by the E-P-OS network confers a clonal growth advantage to fibroid cells by inducing specific mutations in key genes such as MED12 and promoting genomic instability. This mechanism may also represent a crucial molecular basis for fibroid heterogeneity and recurrence.

The persistent activity of the E-P-OS network may represent a potential mechanism underlying the enlargement of fibroids, the hardening of their texture, and clinically refractory abnormal uterine bleeding. This also offers a potential explanation for the high recurrence risk patients face after receiving hormone therapy alone or undergoing surgery. However, it must be emphasized that the hypoxic responses, metabolic reprogramming, and accumulation of mutations potentially induced by this network draw upon concepts from certain areas of cancer research. Uterine fibroids are benign tumors exhibiting self-limiting growth without invasive or metastatic potential. These concepts are employed herein to describe biological similarities in local growth, fibrosis, and vascular support, not to imply a tendency toward malignant transformation. Future research should further elucidate the specific regulatory networks governing these mechanisms within the context of benign tumors.

## 4. Clinical translation strategies

**Figure 5.**
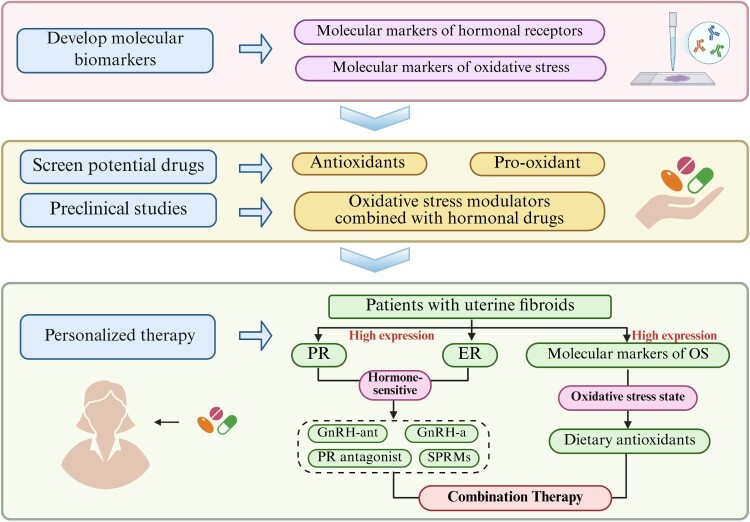
Roadmap for clinical translation strategy. Created in BioRender.

The interaction between hormones and OS within the E-P-OS network may be a key factor contributing to the high incidence and recurrence rates observed in patients with uterine fibroids. Therefore, targeted intervention at critical junctions along the E-P-OS network may resolve this clinical challenge. Future research should focus on the following areas to advance clinical translation. First, develop molecular biomarkers associated with the E-P-OS network to enable precise patient stratification based on the molecular biomarker expression profiles of uterine fibroid patients. Second, screen for potential drugs that modulate OS, advance preclinical studies of combined hormone and anti-OS intervention strategies, and validate their synergistic therapeutic efficacy and safety. Building on this foundation, formulate personalized treatment strategies, drive the translation of relevant findings into clinical applications, and ultimately achieve effective control of uterine fibroid incidence and recurrence rates (Figure 5).

### 4.1. Develop molecular biomarkers

#### 4.1.1. ER and PR

ER and PR, as core molecules regulating E₂ and progesterone sensitivity in uterine fibroids, have been thoroughly validated for their clinical significance through extensive basic research and multicenter clinical practice. In clinical practice, differentiating uterine fibroids from uterine leiomyosarcomas remains a persistent challenge in gynecological pathology and clinical management. Although both originate from uterine smooth muscle tissue, their distinct benign and malignant natures directly influence the determination of surgical approaches and the selection of postoperative adjuvant treatment strategies [116]. Conventional imaging studies are often limited in distinguishing between the two due to overlapping characteristics. However, immunohistochemical detection of ER and PR, with its high sensitivity and specificity, has become an important adjunct to morphological diagnosis, providing key molecular evidence for differentiating uterine fibroids from leiomyosarcoma [117]. Studies consistently demonstrate that ER and PR expression levels in uterine fibroids are significantly higher than in leiomyosarcoma. Approximately 65% of leiomyosarcomas completely lack ER expression, and 75% of leiomyosarcomas lack PR expression. Even when partial ER and PR expression is present in leiomyosarcomas, both the positive intensity and the proportion of positive cells are significantly lower than in uterine fibroids [118]. Additionally, the expression status of ER and PR provides valuable guidance for hormone therapy in uterine fibroids. Patients with high ER and PR expression demonstrate higher response rates to hormone therapy and achieve more significant tumor shrinkage. Conversely, patients with low ER and PR expression often exhibit poor response to hormone therapy, necessitating timely adjustment of treatment regimens [119]. It is noteworthy that high expression of ER and PR also constitutes an independent risk factor for postoperative recurrence, necessitating enhanced postoperative monitoring and adjuvant therapy [11]. Therefore, these patients require enhanced postoperative follow-up monitoring and consideration of adjunctive interventions to reduce recurrence risk. Concurrently, continuous monitoring of changes in ER and PR expression levels serves as an effective indicator for assessing uterine fibroids’ responsiveness to endogenous E_2_ and progesterone. This facilitates more accurate assessment of disease progression trends, enabling physicians to develop personalized treatment and management plans for patients.

#### 4.1.2. OS-related molecular markers

ROS are core initiators of OS in cells. Excessive ROS can cause severe oxidative damage to biomolecules such as DNA, proteins, and lipids within cells [120,121]. Regarding DNA, ROS can induce base oxidation, DNA strand breaks, and gene mutations. 8-OHdG, a key oxidative damage biomarker produced when ROS attack DNA, exhibits significantly elevated levels in uterine fibroid tissue. This directly demonstrates that OS has caused substantial damage to genetic material [122]. 8-OHdG can be detected in urine samples from patients with uterine fibroids. E_2_ and progesterone fluctuate cyclically with the female menstrual cycle. Studies indicate that 8-OHdG levels also significantly increase during the luteal phase of the menstrual cycle, suggesting that E_2_ and progesterone may synergistically exacerbate oxidative damage through 8-OHdG [123]. More importantly, the persistent DNA damage environment induced by 8-OHdG is considered a major driver of the high frequency of MED12 gene mutations [112]. LI et al. investigated the effects of racial differences on the transcriptome and ROS load in uterine fibroids, also finding elevated levels of 8-OHdG in fibroid tissue [17]. Beyond direct damage markers, intracellular antioxidant and repair systems also exhibit corresponding changes. 8-oxoguanine DNA glycosylase (OGG1), a key repair enzyme that specifically recognizes and excises 8-OHdG, is upregulated in uterine fibroids to counteract increased oxidative damage. Concurrently, the expression levels of heme oxygenase-1 (HO-1) and kelch-like ECH-associated protein 1 (KEAP1) are also significantly elevated [17]. Under pathological conditions of uterine fibroids, the significant upregulation of OGG1, HO-1, and KEAP1 expression does not indicate weakened OS but rather represents compensatory activation. This demonstrates that cells are actively resisting oxidative damage and initiating repair mechanisms, thereby corroborating the presence and intensity of OS from another perspective. These genes, together with 8-OHdG, can serve as molecular markers for OS. Regarding proteins, the OS status of proteins in the serum of women with uterine fibroids is significantly heightened. Compared to healthy women, patients with uterine fibroids exhibit markedly elevated levels of protein carbonyls and advanced oxidation protein products (AOPPs) in serum, while thiol levels are significantly reduced. These alterations serve as effective biomarkers reflecting OS status [124]. Regarding lipids, ROS can induce lipid peroxidation. Patients with uterine fibroids commonly exhibit elevated levels of lipid hydroperoxides and reduced antioxidant levels [67]. 8-epi-prostaglandin F2α (8-epi-PGF2α) is considered the gold standard marker for lipid peroxidation in vivo. Expression levels of 8-epi-PGF2α are significantly higher in patients with uterine fibroids than in healthy women [123].

Based on the interactions between hormones and the OS within the E-P-OS network, Table 1 lists representative key molecular markers that directly reflect hormone sensitivity and OS status in uterine fibroid patients. By assessing the expression levels of these markers, patients can be classified into hormone-sensitive and OS-dominant types. This classification provides a basis for developing personalized treatment strategies.

**Table 1.** Molecular biomarkers for stratified diagnosis and therapy in patients with uterine fibroids.

Molecular markers	Representative type	Main sources of samples	Clinical significance
Molecular markers of hormonal receptors			
ER	Estrogen receptor overexpression	Uterine fibroid tissue	Estrogen sensitivity↑
PR	Progesterone receptor overexpression	Uterine fibroid tissue	Progesterone sensitivity↑
Molecular markers of OS			
8-OHdG	DNA oxidative damage	Urine	OS and DNA oxidative damage↑
OGG1	DNA oxidative damage	Uterine fibroid tissue	OS and DNA repair capacity↑
HO-1	Redox imbalance	Uterine fibroid tissue	OS↑
KEAP1	Redox imbalance	Uterine fibroid tissue	Nrf2 pathway activation↑
Protein carbonyls	Protein oxidation	Serum	OS and protein oxidation↑
AOPPs	Protein oxidation	Serum	OS and protein oxidation↑
Thiols	Protein oxidation	Serum	OS and protein oxidation↑
8-epi-PGF2α	lipid peroxidation	Urine	OS and lipid peroxidation↑

#### 4.1.3. Screen potential drugs for clinical translation

The existence of the E-P-OS network makes simultaneously improving hormonal imbalance and OS status a highly promising therapeutic strategy for treating uterine fibroids. Currently, hormone therapy has reached relative maturity in clinical practice, while treatments targeting OS remain in the exploratory phase. It is well known that antioxidants can effectively improve OS levels [125]. Based on this, we systematically reviewed and screened antioxidants with potential anti-fibroid activity. Concurrently, we discovered that certain non-antioxidant compounds can also inhibit fibroid growth by influencing OS levels. Research confirms that these OS modulators demonstrate significant therapeutic efficacy across key therapeutic targets, including inhibiting abnormal proliferation of uterine fibroid cells, inducing apoptosis, reducing tissue fibrosis, and suppressing pathological angiogenesis. Based on the core functional characteristics of these intervention agents, we categorize them into antioxidants and non-antioxidants to summarize relevant therapeutic targets. This provides a clear classification framework and potential directions for subsequent mechanism studies and clinical translation.

#### 4.1.4. Antioxidants

Resveratrol is a natural polyphenolic compound widely found in plants such as *Polygonum cuspidatum* and grapes. Its prominent antioxidant properties can reduce OS-induced cellular damage by scavenging excess free radicals in the body [126]. CHEN et al. found that resveratrol reduces the expression levels of ECM-related proteins such as COL1A1, FN1, α-smooth muscle actin (α-SMA), and β-catenin in primary human leiomyoma cells, thereby inhibiting fibrotic changes in leiomyomas. Animal studies demonstrate that resveratrol significantly suppresses uterine fibroid growth by inhibiting cell proliferation and promoting apoptosis [127]. Luteolin is a flavonoid found in vegetables, fruits, and plants, exhibiting both antioxidant and anti-inflammatory properties [128]. BINMAHFOUZ et al. found that luteolin reduces uterine weight, exhibits pro-apoptotic and anti-proliferative effects, improves the proliferation of fibroid cells in a rat uterine fibroid model induced by diethylstilbestrol and progesterone, significantly lowers α-SMA protein expression, and inhibits TGF-β1 and PI3K-mediated signaling pathways by enhancing PTEN expression, ultimately achieving an anti-fibroid growth effect [129]. *Myrianthus arboreus* is a plant native to tropical regions of Africa, and most studies indicate it possesses antioxidant properties [130,131]. ATEBA et al. found that *Myrianthus arboreus* extract exhibits antioxidant effects by reducing malondialdehyde levels and increasing catalase and GSH activity, while also lowering serum E_2_ levels in rat models [132]. Additionally, studies revealed that *Myrianthus arboreus* inhibits inflammation, fibrosis, and angiogenesis in fibroid tissue by reducing TGF-β1, VEGF, and TNF-α levels, thereby suppressing fibroid growth [132]. *Polyscias fulva* is a plant species belonging to the genus *Polyscias* within the family Araliaceae. It has been demonstrated to possess antioxidant properties and to treat uterine fibroids by reducing serum E_2_ and progesterone levels [133,134]. KIYIMBA et al. demonstrated through network pharmacology, molecular docking, molecular dynamics simulations, and in vivo analyses that *Polyscias fulva* inhibits uterine fibroid growth. The study suggests *Polyscias fulva* may exert its effects by regulating the *HIF1A*, *ESR1*, *EGFR*, and *CASP3* genes, promoting caspase-3 expression, and suppressing HIF-1α, ERα, and EGFR expression [135].

Licochalcone A, nerolidol, and deoxyelephantopin are natural plant compounds widely distributed in plants such as *Glycyrrhiza glabra*, *Santalum album, and Elephantopus scaber*. Due to their potent antioxidant properties, they are applied in the treatment of skin disorders, metabolic diseases, and malignant tumors [136,137,138]. However, current research indicates that during the treatment of uterine fibroids, they enhance OS by generating large amounts of ROS, exhibiting pro-oxidative characteristics that induce apoptosis in fibroid cells under high OS conditions. CHIEN et al. found that licochalcone A promotes ROS production in a dose-dependent manner via the JNK/GRP78/NRF2 signaling pathway. This ROS-induced endoplasmic reticulum stress significantly increases protein expression, induces apoptosis in ELT3 cells, and reduces tumor growth and weight in nude mouse models [139]. DONG et al. found that nerolidol induces cellular DNA damage by promoting ROS production and inhibiting the Akt pathway. It downregulates the expression of cyclin D1, cyclin-dependent kinase (CDK) 4, and CDK6 proteins in a dose-dependent manner, arresting the cell cycle at the G1 phase. This mechanism achieves the suppression of ELT3 cell proliferation [140]. PANDEYDOE et al. discovered that deoxyelephantopin induces mitochondrial membrane potential depolarization, increases ROS accumulation, and induces OS, leading to cell cycle arrest at the G2/M phase. This inhibits the growth of uterine leiomyoma cells, promotes caspase-3 and Bax expression, and suppresses Bcl-2 expression to induce apoptosis [141].

It should be noted that strawberries possess high antioxidant capacity due to their rich anthocyanin content. Strawberry extract demonstrates antioxidant effects by reducing ROS concentration and enhancing the vitality of normal uterine myometrial cells. Conversely, in uterine fibroids, it induces a pro-oxidative state characterized by elevated intracellular ROS levels, thereby promoting fibroblast apoptosis and reducing glycolysis and fibrosis. Consequently, strawberry extract exhibits bidirectional regulatory effects on both normal and abnormal cells [142]. GIAMPIERI et al. investigated the effects of different strawberry cultivars on uterine fibroids, finding Romina to be the most effective cultivar, followed by Alba [142]. Romina and Alba anthocyanin extracts reduce fibrosis in uterine fibroid cells and inhibit ECM components such as FN, COL1A1, and Activin A. Among these, Alba anthocyanin also inhibits versican and plasminogen activator inhibitor-1 (PAI-1) [143]. These findings suggest that strawberry extract possesses excellent OS-modulating capabilities and may be developed as a therapeutic or preventive agent for uterine fibroids.

#### 4.1.5. Non-antioxidants

In addition to antioxidants, other interventions may also inhibit uterine fibroid growth by elevating OS levels through increased ROS production. JOUNG et al. discovered that the proteasome inhibitor MG132 promotes ROS-dependent apoptosis in ELT3 cells by increasing ROS levels in a concentration- and time-dependent manner. The molecular mechanism involves regulating the activity of proteins such as p21, p27, ERK, and caspase-3, while simultaneously promoting the conversion of microtubule-associated protein 1 light chain 3(LC3)-I to LC3-II to activate autophagy [144]. Nicotinamide phosphoribosyltransferase (NAMPT) is the key rate-limiting enzyme in nicotinamide adenine dinucleotide (NAD) synthesis, influencing cellular metabolism by regulating NAD regeneration [145]. CHIANG et al. emphasized the pivotal role of NAMPT, NAD metabolism, and redox signaling in uterine fibroids. Elevated NAMPT levels positively correlated with increased ECM deposition and expression of desmoplastic markers. Treatment of ELT3 cells with the NAMPT inhibitor FK866 significantly elevated OS levels, inducing mitochondrial dysfunction and DNA damage. This modulated cellular stemness by inhibiting cell proliferation and reducing fibrosis through cell cycle arrest [146]. LIN et al. discovered that the alkaloid Manzamine A targets sterol O-acyltransferase (SOAT), downregulating FN, MMP-2, and MMP-9, while inducing mitochondrial oxidative phosphorylation dysfunction and ROS accumulation. This triggered G0/G1 phase arrest, endoplasmic reticulum stress, and caspase-3-mediated apoptosis in ELT3 cells [147]. LEE et al. found that Gyejibongnyeong-Hwan (GBH) induces apoptosis by elevating mitochondrial ROS levels, promoting p53, caspase-3, and caspase-9 expression, and increasing the Bax/Bcl-2 ratio, demonstrating GBH's potential as a therapeutic agent for uterine fibroids [95]. Metformin, a drug widely used in the treatment of type 2 diabetes, has garnered significant attention in recent years for its mechanisms of action and therapeutic potential in regulating overall survival [148]. Metformin has been explored for the treatment of various diseases due to its ability to reduce ROS production and activate the antioxidant system [149,150]. TADAKAWA et al. discovered that Metformin inhibits VEGF protein levels in ELT3 cells in a dose-dependent manner. Particularly under hypoxic conditions, it also suppresses HIF-1α protein expression, thereby inhibiting VEGF activity through the mTORC1/HIF-1α pathway and exhibiting anti-angiogenic effects [151].

In summary, these OS modulators can influence OS status by regulating ROS, thereby achieving therapeutic effects on uterine fibroids through either inhibiting or promoting OS. In Table 2, we have provided a detailed list of specific information, such as the intervention concentrations and durations corresponding to different reagents, with the aim of offering comprehensive and valuable references for subsequent studies. Future research may focus on conducting preclinical experiments combining existing hormonal drugs with OS modulators, thereby establishing a robust experimental foundation for clinical translation.

**Table 2.** Targets and effects of OS modulators on uterine fibroids.

Oxidative stress modulators	Research model	Intervention time	Intervention concentration	Key targets	Efficacy	References
Antioxidants						
Resveratrol	Uterine leiomyoma tissues from patients	48, 72h	0, 10, 50, 100μM	↓: COL1A1, FN1, α-SMA, β-catenin	Induces apoptosis, cell cycle arrest, and and reduces fibrosis.	[127]
	Female nude (Foxn1nu) mice	4w	10 mg/kg(twice a week)	↑: Bax/Bcl-2 ↓: α-SMA, PCNA, FN1	Inhibits fibroid growth.	
Luteolin	Female adult Wistar rats	5w	10 mg/kg/d	↑: PTEN ↓: α-SMA, TGF-β1, PI3K	Inhibits fibroid growth.	[129]
*Myrianthus arboreus*	Female Wistar rats	30d	50, 100, 200 mg/kg/d	↓: TGF-β1, VEGF, TNF-α	Inhibits fibroid growth.	[132]
*Polyscias fulva*	Virgin female Wistar albino rats	14d	200, 400, 800 mg/kg/d	↑: Caspase-3 ↓: HIF-1α, ERα, EGFR	Inhibits fibroid growth.	[135]
Licochalcone A	ELT3	24h, 48h	0, 10, 20, 30, 40, 50, 60μM	↑: Caspase-3, Caspase-9, PARP, GRP78, JNK, p-NRF2/NRF2	Induces apoptosis.	[139]
	BALB/c nude mice	28d	0, 10, 20 mg/kg	↑: JNK, NRF2, GRP78, Caspase-3	Inhibits fibroid growth.	
Nerolidol	ELT3	48h	0, 25, 50, 100μM	↓: Akt, Cyclin D1, CDK4, CDK6	Induces cell cycle arrest and inhibits proliferation.	[140]
Deoxyelephantopin	Uterine leiomyoma tissues from patients	24h, 48h	0, 5, 10, 25μM	↑: Caspase-3, Bax ↓: Bcl-2	Induces apoptosis and cell cycle arrest.	[141]
Romina anthocyanin	Uterine leiomyoma tissues from patients	48h	250μg/ml	↓: FN, COL1A1, Activin A	Induces apoptosis and reduces fibrosis.	[142]
Alba anthocyanin	Uterine leiomyoma tissues from patients	48h	250μg/ml	↓: FN, COL1A1, Activin A, Versican, PAI-1	Induces apoptosis and reduces fibrosis.	[143]
Non-antioxidants						
MG132	ELT3	24h, 48h	0, 0.25, 0.5, 1, 2µM	↑: p21(24h), Caspase-3, LC3 II/I ↓: p21(48h), p27, ERK	Induces apoptosis and triggers autophagy.	[144]
FK866	ELT3	48h	0, 100, 1000nM	↓: NAMPT, PI3K, p-AKT/AKT, Cyclin D1, COL1A1, FN, α-SMA, p-JNK/JNK, CD44, OCT-4	Inhibits proliferation, fibrogenesis, and stemness.	[146]
Manzamine A	ELT3	24h, 48h	2.5, 5μM	↑: Caspase-3, p-53, PARP, Bax/Bcl-2 ↓: SOAT-2, β-catenin, FN, MMP-2, MMP-9	Inhibits proliferation, promotes apoptosis, and reduces fibrosis.	[147]
	BALB/c mice	10d	2 mg/kg/d	/	Inhibits fibroid growth.	
GBH	Uterine leiomyoma tissues from patients	48h	0, 50, 100, 200μg/ml	↑: Bax/Bcl-2, p53, Caspase-3, Caspase-9	Induces apoptosis.	[95]
Metformin	ELT3	48, 72h	0, 0.5, 1, 2.5μM	↓: VEGF, HIF1-α, mTORC1	Inhibits hypoxia-induced angiogenesis.	[151]

### 4.2. Clinical treatment strategies

#### 4.2.1. Hormone therapy

The core strategy of hormone therapy involves inhibiting the signaling pathways of E₂ and/or progesterone through various mechanisms. From the perspective of the E-P-OS network, these therapies primarily intervene in the ‘hormone-driven’ aspect of this network. We summarize the efficacy, limitations, and impact on the E-P-OS network of the following drugs in Table 3.

**Table 3.** Evaluation of hormonal therapies for uterine fibroids.

Drug types	Representative drugs	Mechanism of action	Efficacy	Recurrence and limitations	Impact on the E-P-OS network	References
GnRH-a	Leuprorelin, Goserelin, Triptorelin	Downregulate pituitary function, significantly reducing E₂ and progesterone levels.	Reduces fibroid volume and controls bleeding preoperatively.	Fibroids recur after stopping the medication; menopausal symptoms limit long-term use.	Deprives network of core driver signal via systemic E₂ and progesterone suppression.	[152–155]
GnRH-ant	Elagolix, Relugolix	Blocks GnRH receptor competitively, leading to rapid suppression of E₂ and progesterone.	Effectively controls heavy menstrual bleeding; combined with add-back therapy to mitigate bone loss.	Symptom recurrence after discontinuation; long-term safety of combination therapy under evaluation.	Deprives the network of core driver signal via systemic E₂ and progesterone suppression.	[158–164]
PR antagonist	Mifepristone	Competitive binding and blocking of PR.	Reduces bleeding and inhibits fibroid growth.	Optimal dosage and long-term management strategy remain unclear; significant interindividual variation in efficacy.	Blocks the tumor-promoting effect of progesterone.	[167–169]
SPRMs	UPA	Organizations selectively regulate PR.	Reduces fibroid volume and controls symptoms	Requires monitoring for hepatotoxicity. Long-term endometrial effects require further study.	Modulates progesterone-induced tumor promotion in a tissue-specific manner.	[170–175]

GnRH agonists (GnRH-a) are currently the most widely used hormonal drugs for the clinical treatment of uterine fibroids. Representative drugs include leuprolide, goserelin, and triptorelin. GnRH-a directly inhibits E_2_ production. Furthermore, clinical trials have demonstrated that GnRH-a reduces ER and PR expression in a dose-dependent manner and suppresses vascular endothelial cell proliferation, thereby reducing fibroid growth [152]. Multiple studies have confirmed the significant efficacy of GnRH-a in treating symptomatic uterine fibroids. A double-blind randomized controlled trial demonstrated that GnRH-a effectively reduces intraoperative bleeding, showing superior efficacy compared to ulipristal acetate (UPA) [153]. Meta-analysis results indicate that patients undergoing laparoscopic myomectomy with GnRH-a pretreatment experienced an average reduction of 23.03 mL in intraoperative blood loss, while open surgery demonstrated a more significant reduction of 97.39 mL [154]. Additionally, preoperative administration of GnRH-a reduces uterine volume and fibroid size while increasing preoperative hemoglobin levels [155]. Therefore, preoperative pretreatment with GnRH-a reduces fibroid volume and decreases the formation and number of intraglandular vessels, offering significant advantages in reducing intraoperative blood loss during myomectomy and shortening surgical duration. However, the low E_2_ state induced by GnRH-a often leads to perimenopausal symptoms such as hot flashes, night sweats, insomnia, and vaginal dryness in patients, and may even cause bone loss. These symptoms severely impact patients’ quality of life. Therefore, treatment duration is typically recommended to be limited to 3–6 months [156].

GnRH antagonists (GnRH-ant) represent a novel class of drugs for treating uterine fibroids. Their representative agents, elagolix and relugolix, are both orally administered non-peptide GnRH-ant. Compared to GnRH-a, GnRH-ant causes a sharp decline in serum E_2_ and progesterone levels. This deprives ER and PR of the ligands required for their activation, significantly weakening the effects of the hormonal signaling pathway [157]. Moreover, GnRH-ant demonstrates superior efficacy in reducing menstrual blood loss, making it suitable for patients requiring rapid symptom relief [158,159]. Elagolix and relugolix have been approved for treating abnormal uterine bleeding associated with uterine fibroids. A multicenter Phase III clinical trial confirmed that elagolix significantly reduces menstrual blood loss associated with uterine fibroids, with efficacy unaffected by age, body mass index, ethnicity, baseline bleeding volume, or fibroid location [160]. Combined hormone reversal therapy (elagolix 300 mg/estradiol 1 mg/norethindrone acetate 0.5 mg) mitigated the low estrogen effect of elagolix, demonstrating superior efficacy in preventing bone density decline [161]. The Phase III clinical trial of LIBERTY also confirmed that the combination therapy of relugolix (relugolix 40 mg/estradiol 1 mg/norethindrone acetate 0.5 mg) significantly reduced severe menstrual bleeding associated with uterine fibroids and improved anemia [162]. Relugolix combination therapy maintained symptom relief over 52 weeks, with most patients showing no clinically significant decline in bone mineral density [163]. Notably, after two years of combined treatment with relugolix, patients maintained low menstrual flow, demonstrating the sustained efficacy of this therapeutic approach [163]. GnRH-ant, primarily elagolix and relugolix, can rapidly alleviate symptoms of heavy bleeding. Their combination therapy provides sustained efficacy after discontinuation, offering a non-surgical option for patients wishing to preserve fertility [164]. However, although combined reverse administration may mitigate the side effects of low E_2_, long-term safety data remain limited [165], and as a novel drug, it may face higher cost issues [166].

Progesterone receptor antagonists (PR antagonists) exert their effects by competitively binding to the PR, thereby blocking the biological effects of progesterone. This mechanism inhibits fibroid growth and alleviates clinical symptoms. Mifepristone, the earliest PR antagonist studied for uterine fibroid treatment, exhibits potent anti-progesterone activity. Currently, numerous clinical trials have been conducted to evaluate the efficacy and safety of mifepristone in treating uterine fibroids. A prospective interventional study suggests that perimenopausal women with uterine fibroids who took 25 mg of mifepristone daily in consecutive cycles of 3 months each, for a total of 2–4 cycles, experienced reduced fibroid volume and increased hemoglobin levels after 12 months [167]. Another prospective study also demonstrated that 25 mg mifepristone significantly reduces bleeding, inhibits fibroid growth, and shrinks their volume. With its practicality, cost-effectiveness, and convenience, it serves as a viable alternative to surgery [168]. A meta-analysis evaluated the safety and efficacy of different doses for treating uterine fibroids, indicating that 5 mg/day may be the optimal clinical dose. This dosage effectively reduces fibroid volume while minimizing the risks of endometrial thickening and elevated liver transaminases [169]. Based on robust clinical research findings, mifepristone has been approved in multiple countries for preoperative treatment of uterine fibroids or long-term management of patients unsuitable for surgery. Clinical practice increasingly favors low-dose mifepristone for uterine fibroids, as it delivers therapeutic efficacy while minimizing adverse reactions. Although mifepristone has demonstrated significant success in treating uterine fibroids, the optimal dosage and treatment duration remain incompletely defined, and individual responses to mifepristone therapy vary.

SPRMs are a class of drugs that specifically target PR, competitively inhibiting the binding of endogenous progesterone to PR and thereby reducing progesterone activity [170]. Compared to traditional PR antagonists, SPRMs offer the most significant advantage of tissue selectivity, enabling targeted therapeutic effects on diseased tissues while minimizing non-specific impacts on healthy tissues. This avoids side effects such as perimenopausal symptoms and osteoporosis associated with conventional drugs, significantly enhancing drug safety and patient tolerance [171]. UPA is the first SPRM approved for the short-term treatment of uterine fibroids. It blocks progesterone's pro-proliferative signaling to fibroid cells, induces fibroid cell apoptosis, and inhibits fibrosis, thereby achieving a significant reduction in fibroid volume [172]. Studies have demonstrated that UPA is an effective therapeutic option, but cases of severe acute drug-induced liver injury have been identified post-marketing, necessitating monitoring of liver function [173,174]. Although UPA was temporarily discontinued, research on uterine fibroids continues. A Phase III randomized controlled trial in Asian populations confirmed the efficacy of UPA in treating menorrhagia, demonstrating comparable results to leuprolide. No significant adverse events were observed with UPA in the study [175]. Another Phase III randomized controlled trial demonstrated that UPA was comparable to the levonorgestrel intrauterine system in improving quality of life, but with a higher rate of amenorrhea. No adverse events such as endometrial malignancies or hepatotoxicity were reported throughout the follow-up period [173]. Current evidence supports the efficacy of UPA in treating uterine fibroids, but more long-term data is needed to confirm its effects on the endometrium and liver function [176].

In summary, hormone therapy effectively controls symptoms in the clinical management of uterine fibroids. However, the high recurrence rate after discontinuation suggests that simply suppressing systemic hormone levels may be insufficient to dismantle the established self-sustaining pathological network within the local microenvironment of the fibroids. This clinical dilemma provides strong indirect support for the E-P-OS network framework. From the perspective of disease progression, the progressive atrophy of fibroids following natural menopause confirms that long-term, complete elimination of hormonal drivers can dismantle this pathogenic network. Conversely, the rapid recurrence observed after all temporary hormonal therapies demonstrates that short-term interventions struggle to achieve comparable outcomes. This clinical evidence collectively supports the notion that the maintenance of uterine fibroids may depend on a stably established E-P-OS network within their local microenvironment, which retains the capacity for self-activation and reconstruction even after short-term hormonal suppression.

#### 4.2.2. Antioxidant therapy

In the field of OS intervention for treating uterine fibroids, drugs represented by dietary antioxidants have demonstrated potential for clinical translation. Among these, vitamin D and (-)-Epigallocatechin-3-gallate (EGCG) are central to ongoing research. Notably, their effects extend beyond simple antioxidant activity, through multi-targeted mechanisms, they offer therapeutic approaches for intervening in the E-P-OS network.

The potential value of vitamin D in treating uterine fibroids primarily stems from its dual properties of antioxidant activity and hormone regulation. Vitamin D exerts extensive genomic effects by binding to the vitamin D receptor (VDR). Defects in VDR function impair its mediation of transcriptional regulation and activation of associated signaling pathways, ultimately leading to uncontrolled cell proliferation [177]. In terms of antioxidant activity, vitamin D not only reduces ROS production but also enhances the activity of endogenous antioxidant defense systems. This effect has been demonstrated in multiple disease models [178,179]. In terms of hormonal regulation, vitamin D can also antagonize the proliferative effects of E₂ and progesterone in uterine fibroid tissue. Specifically, it downregulates the expression of ERα, PR-A, and PR-B, thereby directly interfering with the classical hormone-driven signaling pathways [180]. Therefore, vitamin D may exert a potential dual effect on the OS and hormone signaling pathways within the uterine fibroid E-P-OS network. A case–control study found that serum vitamin D levels in patients with uterine fibroids were significantly lower than in healthy controls, with lower vitamin D levels corresponding to a greater number of fibroids [181]. After three months of daily supplementation with 1600 IU of vitamin D, patients exhibited significantly elevated serum vitamin D levels, along with a marked reduction in both the average diameter and hardness of fibroids. Additionally, symptoms such as dysmenorrhea, urinary frequency, and menorrhagia were significantly alleviated [182]. Multiple clinical studies have confirmed that serum vitamin D levels are negatively correlated with uterine fibroids. Supplementing with vitamin D can significantly inhibit fibroid growth and reduce the risk of recurrence [183]. These clinical data indicate that vitamin D supplementation represents a safe and effective potential treatment option for uterine fibroids.

EGCG is the primary polyphenolic compound in green tea, with its mechanism of action centered on potent antioxidant activity [184]. It not only directly scavenges free radicals but also enhances the function of endogenous antioxidant defense systems by activating pathways such as Nrf2 (Nuclear factor erythroid 2-related factor 2) [185]. However, its effects extend far beyond this. Research indicates that EGCG significantly suppresses mRNA or protein levels of key fibrotic proteins in fibroid cells, thereby inhibiting fibrosis in uterine fibroids [186]. EGCG demonstrates promising potential for clinical translation and may emerge as a safe and effective oral treatment option. A double-blind randomized trial involving 39 symptomatic uterine fibroid patients revealed that daily oral administration of 800 mg green tea extract (containing 45% EGCG) for four consecutive months resulted in a significant 32.6% reduction in fibroid volume, thereby improving biochemical quality of life by alleviating severe symptoms [187]. Combining EGCG with vitamins B and D may yield better results. A study involving 16 patients over 40 with uterine fibroids demonstrated that daily combined supplementation of 300 mg EGCG, 50μg vitamin D, and 10 mg vitamin B6 for 90 days resulted in a significant average reduction of 37.3% in the size of individual fibroids, with particularly favorable outcomes for intramural fibroids. These patients experienced shorter menstrual cycles post-treatment without adverse reactions, offering a new therapeutic option for those unsuitable for hormone therapy [188]. Regarding safety assessment, a prospective study monitored liver function after 3 months of treatment with EGCG combined with vitamin D and D-chiro-inositol, demonstrating favorable surgical outcomes and no hepatotoxicity [189].

Available evidence indicates that vitamin D and EGCG represent a class of therapeutic candidates with multiple mechanisms of action. Vitamin D, in particular, not only alleviates OS but also exerts therapeutic effects by regulating key nodes in hormonal signaling pathways. This multi-targeted mechanism of action positions it as a potential strategy to overcome the limitations of current single-target therapies, offering a new direction for the clinical management of uterine fibroids. However, the optimal dosing regimens, long-term efficacy, and safety of these drugs require further validation through rigorously designed large-scale clinical trials.

#### 4.2.3. Individualized treatment strategies

A wide variety of hormone therapies are currently used clinically to treat uterine fibroids, with each drug offering distinct advantages based on its unique mechanism of action. However, patients exhibit varying hormone receptor sensitivities and OS status, leading to inconsistent treatment outcomes. By detecting relevant molecular biomarkers, implementing stratified treatment approaches, and dynamically adjusting therapeutic regimens, treatment efficacy can be maximized. Uterine fibroids commonly overexpress ER and PR. Hormone therapy should be prioritized during treatment to block the stimulatory effects of E_2_ and progesterone on fibroid cells, thereby inhibiting fibroid growth. GnRH analogues, PR antagonists, and SPRMs all demonstrate effective therapeutic outcomes for fibroids, though their pharmacological mechanisms differ. To achieve personalized drug therapy and optimize treatment efficacy, the expression status of the molecular markers ER and PR can be assessed to guide drug selection. Patients with high expression of both ER and PR demonstrate better response to hormone therapy, particularly those with significantly elevated PR expression who may be prioritized for PR antagonists or SPRMs. These agents can more specifically block PR-related signaling pathways, potentially yielding superior therapeutic responses.

Due to the E-P-OS network present in uterine fibroids, most patients may be in the OS state. Detecting the expression levels of OS-related molecular markers in patients provides compelling evidence confirming their OS status. Existing clinical observations indicate that dietary antioxidants demonstrate certain therapeutic effects in uterine fibroid patients, and supplementing key nutrients (such as vitamin C and vitamin D) can play an adjunctive role [190]. For patients with hormone dependency and high levels of OS, combined hormone and antioxidant therapy may prove a more effective approach. This could involve supplementing conventional hormone treatment with nutrients such as vitamin D. For patients exhibiting low hormone sensitivity but significant OS damage, the focus should shift toward antioxidant therapy while appropriately adjusting the intensity and modality of hormone intervention. Based on current research, this personalized combination approach may theoretically suppress uterine fibroids more effectively while reducing recurrence and adverse reactions. However, preclinical studies are needed to further explore and validate its efficacy and safety in humans. This will better facilitate the translation of basic research findings into clinical applications, offering a practical solution to address the high recurrence rate of uterine fibroids.

## 5. Challenges and future directions

### 5.1. Verification of the E-P-OS network

The E-P-OS network framework provides a valuable integrated perspective for understanding the persistent growth of uterine fibroids. However, the specific mechanisms within this complex network remain to be clearly elucidated.

#### 5.1.1. The evidential strength of the E-P-OS network

Firstly, while the E-P-OS network framework offers an insightful integrated perspective for understanding the persistent growth of uterine fibroids, it is crucial to recognize that the direct experimental evidence supporting this framework remains insufficient. Many of the associations are based on correlations or extrapolations from other disease models. In particular, the pro-oxidative effects of progesterone, although strongly suggested by clinical observations in patients (such as changes in the FORT/FORD ratio), still lack direct mechanistic evidence [70]. The current understanding of progesterone's effects on NADPH supply and mitochondrial dysfunction relies partially on non-myomatous cell models. Therefore, systematically examining these gaps in evidence is crucial for objectively assessing the reliability of this framework and guiding confirmatory studies.

Secondly, there is currently a lack of direct experimental evidence demonstrating a complete bidirectional feedback loop between E₂, progesterone, and OS within uterine fibroids. While evidence supporting hormonal imbalance as a fundamental condition promoting OS is relatively robust, the precise feedback amplification effect of OS on hormonal signaling requires further direct evidence to substantiate. Although an inverse clinical correlation between OS status and ER or PR expression levels has been observed, and some pathways activated by OS intersect with hormone signaling, these associations remain indirect. In uterine fibroid cells, how OS functionally upregulates the transcriptional activity or protein stability of ERα or PR to directly enhance hormone sensitivity remains a core mechanism gap that has not been experimentally confirmed.

Therefore, to achieve the critical leap from correlational recognition to causal demonstration, future research must systematically conduct reverse complementation experiments in uterine fibroid-specific cell or organoid models. Specifically, through exogenous hormone intervention, we should dynamically monitor intracellular ROS production patterns and redox state transitions. Concurrently, by applying controlled doses and durations of OS intervention, we should systematically evaluate changes in phosphorylation levels of key hormone signaling pathways and the transcriptional activity of hormone receptors. This approach can directly verify whether a self-perpetuating feedback loop exists between E₂, progesterone, and OS, thereby progressively constructing a dynamic connectivity map of the E-P-OS interaction network. This provides conclusive mechanistic evidence for their pathophysiological functions.

#### 5.1.2. The precise role and regulatory mechanism of oxidative stress

Multiple studies suggest that OS is closely associated with the development of uterine fibroids. However, existing evidence is largely limited to observational correlations, such as elevated levels of ROS and reduced antioxidant enzyme activity in fibroid tissues. These phenomena alone do not conclusively establish OS as the primary triggering factor for fibroid formation. Currently, multiple lines of evidence support the notion that hormonal imbalance serves as a core driver in the development and progression of uterine fibroids, generating substantial amounts of ROS during the process of promoting abnormal cellular proliferation and metabolism. These ROS not only potentially amplify the original hormone-induced growth signals by acting as secondary amplifiers, but may also create a microenvironment conducive to fibroid growth by serving as permissive factors through persistent low-level oxidative damage. More importantly, existing theories predominantly emphasize the pathological promotion of chronic persistent OS on uterine fibroids, yet fail to fully integrate its concentration-dependent biphasic effects. Some studies in Table 2 confirm that excessively elevated ROS can instead inhibit fibroblast proliferation and induce apoptosis. This phenomenon suggests that the function of the E-P-OS network strictly depends on the precise concentration and duration of ROS exposure. Once its regulatory equilibrium is disrupted, it may trigger entirely opposite biological outcomes. Therefore, defining the critical threshold at which ROS shifts from promoting proliferation to inducing apoptosis in uterine fibroids, and elucidating the roles of estrogen and progesterone in regulating this balance, has become key to understanding the dynamic behavior of this network. Ignoring this complexity may lead to biased clinical intervention strategies. For instance, antioxidant therapy alone may not be suitable for all pathological stages and could obscure its potential double-edged sword effects.

The precise role of OS in uterine fibroids remains unclear, and future research should focus on clarifying its causal status through interventional studies. Clarify whether OS is the initial factor driving the occurrence of the disease, a synergistic factor that accompanies and amplifies hormonal effects, or a metabolic result after abnormal cell proliferation. This fundamental distinction will directly determine whether the core strategy for clinical intervention should be preventive antioxidant therapy or therapeutic blockade of OS signaling pathways. Additionally, real-time monitoring techniques are needed to elucidate the concentration dynamics of reactive oxygen species within fibroid cells, precisely define the threshold at which their effects shift from promoting proliferation to inducing apoptosis, and simultaneously decipher the regulatory roles of E₂ and progesterone in this equilibrium. This will ultimately provide a rational basis for clinical practice. According to the specific redox state of the patient's uterine fibroids, intervention strategies of antioxidant or pro-oxidant therapy can be precisely selected, thereby achieving truly targeted therapy.

Although direct verification of this framework remains challenging, existing research provides strong support for its plausibility. For example, in a rat model of sex hormone-induced uterine fibroids, administration of natural plant extracts with antioxidant activity (such as *Myrianthus arboreus* and *Polyscias fulva*) can inhibit fibroid growth by reducing serum E₂ and/or progesterone levels [132,135]. This finding aligns with the core framework of the E-P-OS network. Clinically, supplementing with dietary antioxidants or short-term hormone therapy may result in an observable reduction in fibroid volume. However, the tendency for recurrence after discontinuing treatment underscores the dynamic and persistent nature of this pathological network. Animal experiments and clinical observations complement each other, jointly establishing the empirical foundation for the E-P-OS network framework and pointing the way for subsequent mechanism exploration.

### 5.2. Clinical translation challenges

Validating the molecular mechanisms of the E-P-OS network is a fundamental prerequisite for its clinical translation. However, translating the theoretical framework of the E-P-OS network into clinical practice also presents multiple challenges.

#### 5.2.1. Detection of molecular biomarkers

In terms of detecting molecular biomarkers, combined analysis of indicators such as ER, PR, 8-OHdG, and 8-epi-PGF2α (Table 1) theoretically enables more precise patient stratification. However, regarding detection accuracy, uterine fibroids exhibit heterogeneity, including internal structural variations, collagen content, and hardness within individual fibroids. This heterogeneity may lead to differences in ER and PR expression levels across different tissue samples [191]. Although 8-OHdG and 8-epi-PGF2α are classic markers of OS, their elevated levels are not specific to uterine fibroids; similar changes can also be observed in various chronic inflammatory diseases and malignant tumors [192,193]. Secondly, regarding sample detection, tissue biopsy currently serves as the gold standard for assessing ER and PR status. However, this invasive procedure limits the strategy's widespread adoption in large-scale population screening and long-term follow-up. Most critically, there remains no universally agreed-upon cutoff value for defining ‘hormone sensitivity’ or ‘high OS status,’ further hindering its standardized application.

To address these transformation challenges, it is recommended to conduct a comprehensive evaluation of fibroids using imaging modalities such as ultrasound or MRI prior to ER and PR testing. Under real-time imaging guidance, precise needle sampling should be performed on regions with distinct characteristics to minimize errors caused by tumor heterogeneity [194]. In the future, actively developing non-invasive diagnostic prediction models for uterine fibroid subtypes based on imaging will help reduce reliance on invasive biopsies [195]. For detecting OS levels, prioritize the development of non-invasive urine-based detection methods [196]. For example, prioritize the use of markers such as 8-OHdG and 8-epi-PGF2α, which are easily collected non-invasively. Building on this foundation, focus on promoting the combined detection and analysis of OS-related markers to enhance the accuracy of molecular subtyping. Finally, through multicenter, large-sample clinical cohort studies combined with statistical methods, derive and validate clinically meaningful cutoff values to enable precise patient stratification and individualized treatment.

#### 5.2.2. Selection of OS-modulating agents

Although the various antioxidants and pro-oxidants listed in Table 2 have demonstrated potential for regulating OS and inhibiting uterine fibroids, the complexity of selecting OS modulators for clinical translation manifests across multiple levels. First, most studies have focused on the tumor-specific effects, and the long-term impact of these modulators on systemic redox balance remains unknown. Long-term use of antioxidants may interfere with normal immune surveillance functions, while pro-oxidants may cause unintended damage to healthy tissues. The complex composition of natural product extracts may induce off-target effects, potentially impacting the function of other organs such as the liver and kidneys. Long-term safety data for these compounds is particularly lacking [197]. Secondly, the effects of most OS modulators exhibit significant context dependency and even contradictory properties. For instance, anthocyanin extracts from ‘Romina’ and ‘Alba’ possess potential bidirectional regulatory capabilities. They can maintain cellular viability by reducing ROS concentrations in normal uterine myometrial cells while simultaneously inducing apoptosis in uterine fibroid cells by elevating intracellular ROS levels to promote OS [142,143]. Traditionally, substances known to have antioxidant properties, such as licochalcone A, nerolidol, and deoxyelephantopin, promote the apoptosis of uterine fibroid cells by increasing ROS [139,140,141]. This paradox in the direction of action is closely related to its inherent chemical properties, the concentration at which it is administered, the duration of its effect, and the pathophysiological state of the cells [198,199]. Finally, most current studies are based on cell lines (such as ELT3) or animal models, whose tumor microenvironments and drug metabolism differ from those in the human uterus. Uterine fibroids exhibit intratumoral and intertumoral heterogeneity, leading to potential variations in therapeutic response to the same intervention across different patients or even different lesions within the same patient. Consequently, the limitations of existing preclinical models constrain the extrapolation of results.

Organoid models are an emerging preclinical research tool in recent years and may solve this problem. They provide a powerful platform for precision medicine and new drug development [200]. Three-dimensional organoid models derived from human uterine myometrial stem cells can mimic the in vivo behavior of uterine fibroids [201]. This means they can more accurately reflect the response of fibroids to hormones, thereby assessing treatment efficacy [202]. Additionally, organoids effectively preserve the cellular diversity of the original tumor during culture, maintaining the heterogeneity of uterine fibroids. This enhances the clinical relevance of research findings [203]. This approach will provide a thorough elucidation of the concentration and time thresholds for drug action, clarify the critical conditions and mechanisms governing its antioxidant or pro-oxidative effects, and systematically evaluate its long-term safety. Consequently, it will furnish precise and reliable experimental evidence for clinical translation, thereby mitigating potential risks arising from improper use.

#### 5.2.3. Safety assessment of combination therapy

The combination of hormone drugs and antioxidants theoretically represents a rational strategy capable of simultaneously disrupting the E-P-OS network at multiple targets. However, this combined approach necessitates careful consideration of critical issues such as drug interactions, additive toxicity, and optimal dosing sequences. However, this combination regimen requires careful consideration of critical issues such as drug interactions, additive toxicity, and the timing of administration. To address the aforementioned issues, clinical trials evaluating the synergistic effects of combined hormone and antioxidant therapies are required. This should involve establishing stable preclinical models to first conduct in vitro experiments for screening combination regimens with potential synergistic effects and low interaction risks. Subsequently, in vivo experiments should validate the efficacy and safety of these regimens within the body while exploring optimal dosing sequences. Therefore, to address this challenge, we can still conduct drug screening and efficacy evaluation using organoid models, combined with cellular and animal experiments, to expedite the identification of effective regimens for the combined use of hormone drugs and antioxidants, while assessing the safety and efficacy of such combination therapies.

## 6. Conclusion

The E-P-OS network framework offers a novel perspective for understanding the persistent growth of uterine fibroids and their recurrence following conventional treatments. The persistent activity of this network is likely a key factor contributing to the enlargement of fibroids, the hardening of their texture, and their tendency to recur after conventional treatments. Therefore, simultaneously targeting hormone-driven mechanisms and OS states to disrupt this self-reinforcing network represents a promising new therapeutic strategy for uterine fibroids. Within this framework, combined therapy involving hormone treatment and antioxidants demonstrates viable therapeutic potential. However, future research must achieve clinical translation through the design of rigorous basic experiments and clinical trials. This will primarily focus on systematically validating the mechanisms underlying the establishment of the E-P-OS network and the efficacy, safety, and long-term therapeutic effects of combined treatment strategies.

**Abbreviations**
4-OH-E₂: 4-Hydroxyestradiol
•OH: Hydroxyl radical
17β-HSD: 17β-hydroxysteroid dehydrogenase
8-epi-PGF2α: 8-epi-prostaglandin F2α
8-OHdG: 8-hydroxy-2'-deoxyguanosine
AMPK: AMP-activated protein kinase
AOPPs: Advanced oxidation protein products
ATP: Adenosine triphosphate
Bax: BCL2-associated X protein
Bcl-2: B-cell lymphoma 2
Bcl-xL: B-cell lymphoma-x
CAT: Catalase
CDK: cyclin-dependent kinase
c-Myc: Cellular myelocytomatosis
COL1A1: collagen type I alpha 1 chain
COL3A1: collagen type III alpha 1 chain
CYP: Cytochrome P450
E_1_: Estrone
E_2_: Estradiol
E₂-3,4-Q: Estradiol-3,4-quinone
ECM: Extracellular matrix
EGCG: (-)-Epigallocatechin-3-gallate
EGF: Epidermal growth factor
EGFR: Epidermal growth factor receptor
E-P-OS: Estrogen-progesterone-oxidative stress
ER: Estrogen receptor
ETC: Electron transport chain
FN: Fibronectin
FORD: Free oxygen radicals defense
FORT: Free oxygen radicals test
GBH: Gyejibongnyeong-Hwan
GnRH: Gonadotropin-releasing hormone
GnRH-a: GnRH-agonist
GnRH-ant: GnRH-antagonis
GRP78: glucose-regulated protein 78
GSH: Glutathione
H₂O₂: Hydrogen peroxide
HIF: Hypoxia-inducible factor
HMGA2: high mobility group AT-hook 2
HO-1: Heme oxygenase-1
IGF-1: Insulin-like growth factor 1
JNK: c-Jun N-terminal kinase
KEAP1: Kelch-like ECH-associated protein 1
LC3: Microtubule-associated protein 1 light chain 3
MAPK/ERK: Mitogen-activated protein kinase/extracellular signal-regulated kinase
MED12: Mediator complex subunit 12
MMPs: Matrix metalloproteinases
NAD: Nicotinamide adenine dinucleotide
NADPH: Nicotinamide adenine dinucleotide phosphate
NAMPT: Nicotinamide phosphoribosyltransferase
NF-κB: Nuclear factor kappa-light-chain-enhancer of activated B cells
NOX: Nicotinamide adenine dinucleotide phosphate oxidase
NRF-1: Nuclear respiratory factor 1
NRF-2: Nuclear respiratory factor 2
Nrf2: Nuclear factor erythroid 2-related factor 2
O₂•^−^: Superoxide anion
OGG1: 8-oxoguanine DNA glycosylase
OS: Oxidative stress
PAI-1: plasminogen activator inhibitor-1
PCNA: Proliferating cell nuclear antigen
PGC-1α: Peroxisome proliferator-activated receptor gamma coactivator 1-α
PI3 K/Akt/mTOR: Phosphatidylinositol 3-kinase/protein kinase B/mechanistic target of rapamycin
PR: Progesterone receptor
PTEN: Phosphatase and tensin homolog
ROS: Reactive oxygen species
SOAT: Sterol O-acyltransferase
SOD: Superoxide dismutase
SPRMs: Selective progesterone receptor modulators
TCA: Tricarboxylic acid
TFAM: Mitochondrial transcription factor A
TGF-β: Transforming growth factor-β
TIMP-1: Tissue inhibitor of metalloproteinase 1
UPA: Ulipristal acetate
VEGF: Vascular endothelial growth factor
VDR: Vitamin D receptor
α-SMA: α-smooth muscle actin

## Data Availability

No data was used for the research described in the article.
